# Cost Analysis of Intranatal Care Services at a Tertiary Care Public Sector Hospital in Rajasthan, India

**DOI:** 10.7759/cureus.41090

**Published:** 2023-06-28

**Authors:** Madhvi Dhamania, Kusum Gaur, Jai Prakash Pankaj, Dharmesh K Sharma, Rajeev Yadav, Dilip Raj

**Affiliations:** 1 Community Medicine, Sawai Man Singh (SMS) Medical College, Jaipur, IND; 2 Public Health, State Institute of Health and Family Welfare, Jaipur, IND

**Keywords:** health economic, hospital cost, normal delivery cost, caesarean section cost, institutional delivery, cost analysis, provider-perspective, bottom-up costing, intra-natal care services

## Abstract

Introduction

India is responsible for the second-highest maternal deaths and the greatest burden of stillbirths worldwide. The cost of intranatal services is an important determining factor, especially in developing countries like India. Most studies report the cost of delivery from the patient’s perspective, but there is a lack of studies from the health system’s perspective. This present study aimed to bridge this gap by estimating the overall and unit costs of various types of deliveries at a tertiary-level hospital in Rajasthan, India.

Methods

The cost estimation of intranatal services was conducted in a tertiary-level teaching hospital in Jaipur, Rajasthan. This cost analysis undertook the health system's perspective, using bottom-up costing methodology. Data on all the resources (capital/recurrent) used for the delivery of intranatal care from April 2020 to March 2021 were collected. Sensitivity analysis was done to account for any variability in cost components on overall intranatal service cost.

Results

The annual cost of intranatal care services at the tertiary care hospital was INR 149,011,957 (USD 1,988,152). The unit cost per vaginal delivery was INR 8,244.4 (USD 109.9) and the unit cost per cesarean section was INR 10,696.2 (USD 142.7). Among various heads of expenditure, 'human resource' costs were predominant, accounting for 47.7% of the total costs, followed by 'building/space' and 'overhead' costs, accounting for 30.59% and 11.1%, respectively.

Conclusion

The results may help plan and manage intra-natal care services in Rajasthan. Apart from the judicious utilization of resources, the findings of the study may also serve as a basis for future health economic studies.

## Introduction

Low- and lower-middle-income countries account for 94% of all maternal deaths globally [1]. The impact of the economy on maternal health is evident from the vast disparities in maternal mortality between rich and poor countries [1]. The impact of maternal health on the economy is evident from the reduction of per capita gross domestic product (GDP) by USD 0.36 per year with every maternal death [2].

India is responsible for the second-highest number of maternal deaths as well as the greatest burden of stillbirths worldwide [3,4]. Intrapartum stillbirth is a sensitive marker of the timeliness and quality of intrapartum care [3]. Lack of access to and underutilization of intranatal healthcare services are the main reasons for the high maternal and neonatal mortality rates, with cost being an important factor. Though India overall has been witnessing a persistent decline in the maternal mortality ratio (MMR), the state of Rajasthan, with an MMR of 141 maternal deaths/lakh live births, is quite far from achieving the sustainable development goal target of 70 maternal deaths/lakh live births [5,6]. The main strategy for this reduction has been centered on ensuring access to quality, evidence-based maternal health services along the continuum of care through various national programs [7] as well as publically financed health insurance schemes such as *Ayushmaan Bharat* [8,9] and *Chiranjeevi Yojana *[10]. However, for the implementation and maintenance of such schemes, it is essential to have an evidence-based understanding of the distribution of costs involved. From a public health perspective, the proper allocation of resources is essential to ensuring the delivery of accessible and qualified health care, especially when the budget is a big constraint. Moreover, discrepancies between available financing and the actual costs of the services may result in a financial burden on patients by increasing out-of-pocket expenses. 

The available literature, though limited, is either outdated (National Commission on Macroeconomics and Health (NCMH), 2005) [11] or based on financial costs rather than a more detailed economic costing (National Health Accounts Estimates for India (2018-2019)) [12], which limits its application. Several studies and surveys in India have reported the cost of institutional delivery from the patient’s perspective and estimated the out-of-pocket expenditure undertaken by these patients [13,14]. However, there is a lack of evidence on the economic implications from the health system’s perspective. With the objective of reducing this gap in evidence, the present study conducted a cost analysis of various types of costs occurring in the provision of intranatal services from the health system’s perspective at a tertiary-care public hospital.

## Materials and methods

This present study was an institution-based observational study undertaken in a tertiary-care teaching hospital attached to a government medical college in Jaipur, Rajasthan, India. This hospital is dedicated exclusively to obstetric and gynecological services. The study period for this cost study was from October 2021 to November 2022. It was conducted from the health system’s perspective and utilized bottom-up costing methodology [15,16] to collect cost-related data for intranatal services for the financial year (FY) April 2020-March 2021, to encompass all the seasonal variations. 

**Figure 1:**
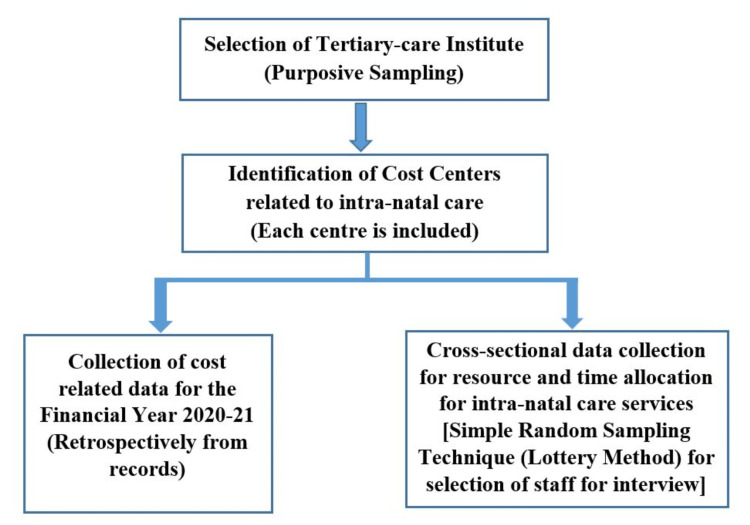
Sampling technique and study design

Purposive sampling was done for the selection of the health care institute, as it caters to large volumes of obstetric patients and is attached to the tertiary-care teaching medical college. A random selection of two personnel for interview from each category of professionals, i.e., paramedical workers, and ancillary staff involved in imparting intranatal care services, was done. They were chosen through a simple random technique-the lottery method. In the event that the personnel were not present on the day of the survey, the next person on the list was chosen. They were interviewed regarding their time allocation to intranatal services exclusively (Figure 1).

After written informed consent was received from the head of the institute, the data collection process was started. All types of costs, direct or indirect, related to intranatal care services were recorded except the costs that were not part of the health system, such as out-of-pocket expenditure, volunteered service costs, and costs related to services linked to complications for the mother and neonate beyond the delivery of a child in the labor room/operation theater.

After the structural organization of the study unit, cost center classification was done. The area of the study unit was divided into primary-care cost centers (PCCs) and secondary-care cost centers (SCCs). The PCCs comprise direct and indirect care centers. The direct care centers include a pre-delivery room (PDR), a clean labor room (CLR), a septic labor Room (SLR), an operation theater (OT), a newborn care corner, and an ICU. The indirect care centers include the radiology department (only for USG) and laboratory (pathology, biochemistry, and microbiology departments). The SCC incorporates water, electricity, laundry, dietetics, administration, security, transport, a blood bank, a pharmacy, maintenance, and miscellaneous units. The types of resources consumed and services rendered at each of these centers were identified, measured, and ultimately categorized under the sub-headings of ‘capital costs’ (building/space and equipment) and ‘recurrent costs’ (human resources, medicines, stationery, consumables, and overheads). Data on the number of vaginal deliveries, cesarean sections, and diagnostic tests were collected. Data on the number of consumables and non-consumables used, the quantity of equipment, their year of purchase, and their price were obtained. Also, a record review of the respective stock registers and account books was done for each cost center. This was triangulated with interviews with the staff and observation of the facility by the investigator. Data on staff salaries and other utilities (water bills, electricity bills, telephone bills, miscellaneous expenditures, etc.) were collected from the hospital administration department. A pre-designed, pre-tested, semi-structured pro-forma that has already been used in previous Indian studies [17,18] was used to collect the relevant data after modifying it for intranatal services. Data on the average life of the equipment were collected by interviewing staff members working with that equipment. The costs of equipment were also obtained from local distributors. Average prevailing market prices for the year 2020-2021 were used. The costs of all recurrent resources, like medicines and consumables, were obtained from the rate contract list of the state government.

Data analysis

Costs were expressed as total annual cost and unit cost in INR and USD for all the outcome variables. All the costs were converted to USD for comparability at the global level at the rate of USD 1 = INR 74.95 (FY 2020-2021) [19]. For the building or space costs, the rental price was multiplied by the floor area to estimate the opportunity cost of the building being used [15]. In cases of joint or shared costs, either capital or recurrent, the cost of the overall resource that was used for intranatal care was apportioned using suitable apportioning statistics, e.g., ‘personnel’ was apportioned as per work time, ‘building’ as per floor area used, and ‘equipment’ as per the time used in intranatal service. The costs of medicines and consumables were calculated by applying the price to the quantity of resources consumed. The annualized cost of other capital resources was estimated considering the life of the item and a discount rate of 5% [20]­­.

Sensitivity analysis

A sensitivity analysis of the total cost to various types of costs related to intranatal care services was done to make it comparable with other regions and times and account for any costing data fluctuations and assumptions. It was also done to identify the key cost drivers affecting the total costs. For analysis, the outcome variables were varied by 25% on either side of the base value and depicted via a tornado diagram.

Ethics statement

The study was approved by the Institutional Ethical Committee of Sawai Man Singh (SMS) Medical College (approval no. 977/MC/EC/2021). Written informed consent was obtained from participants prior to the interview for time allocation. The identity of participants was kept confidential with anonymous entries and by not disclosing their names at any point of the study, including reports. 

## Results

**Figure 2:**
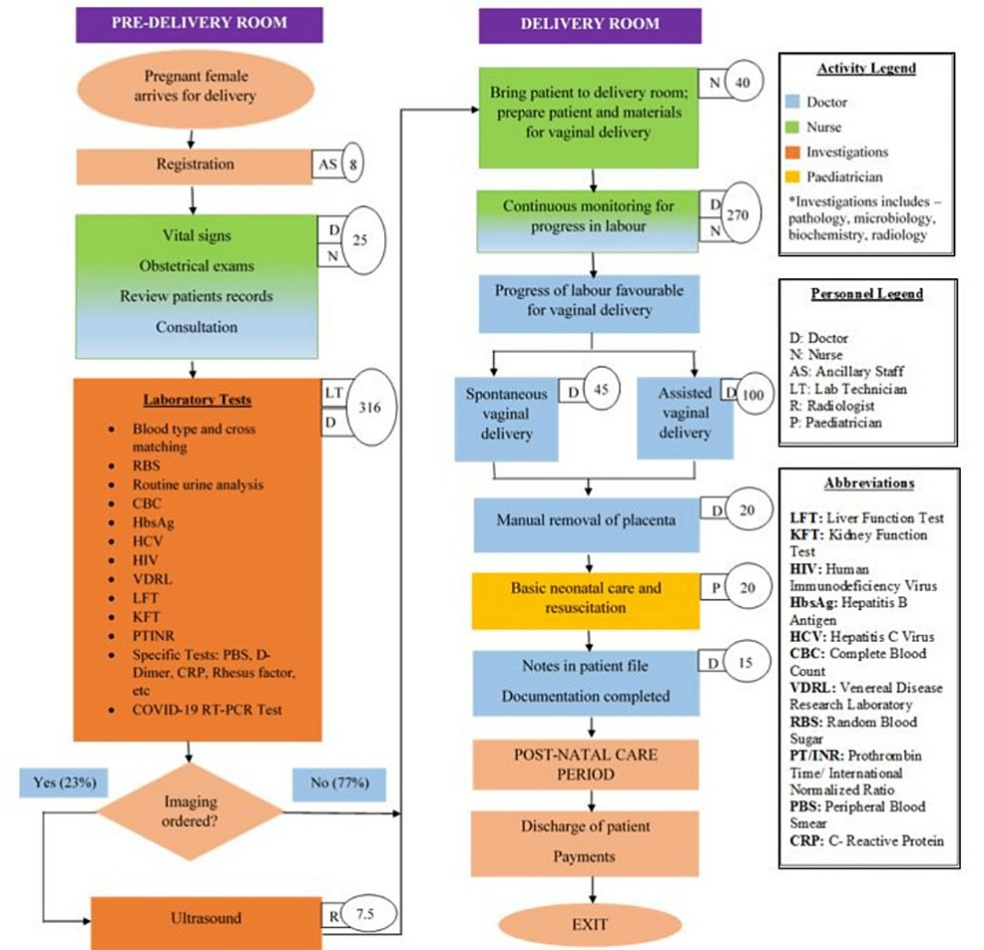
Process map for vaginal delivery

**Figure 3:**
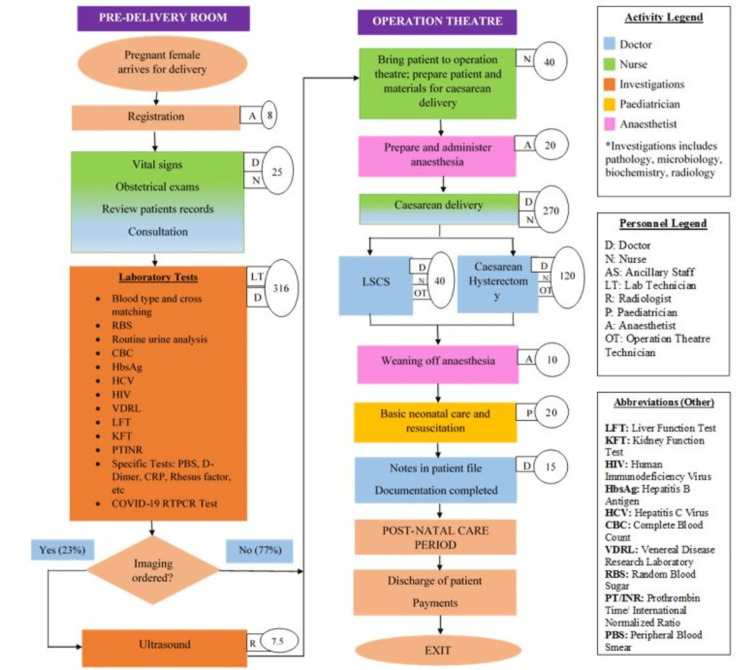
Process map for caesarean delivery

A total of 11,718 women availed of intranatal care services in the financial year 2020-2021. Amongst them, 11,208 (95.65%) were live birth deliveries, 39 (0.35%) were stillbirths, and 471 (4.02%) were intrauterine fetal deaths (IUFD). Among the live births, 5,578 (49.8%) were vaginal deliveries, and 5,630 (50.2%) were caesarean sections. Process maps to depict both these modes of delivery are illustrated in Figures 2-3. The large boxes depict activities, arrows represent the direction of events, small boxes depict the providers involved, and small ovals depict time in minutes spent on every activity. Time displayed is the average of the times reported from interviews of two medical personnel who supervise, perform, or assist that task. This depiction was necessary as it has implications for resource use, and hence its cost.

**Figure 4:**
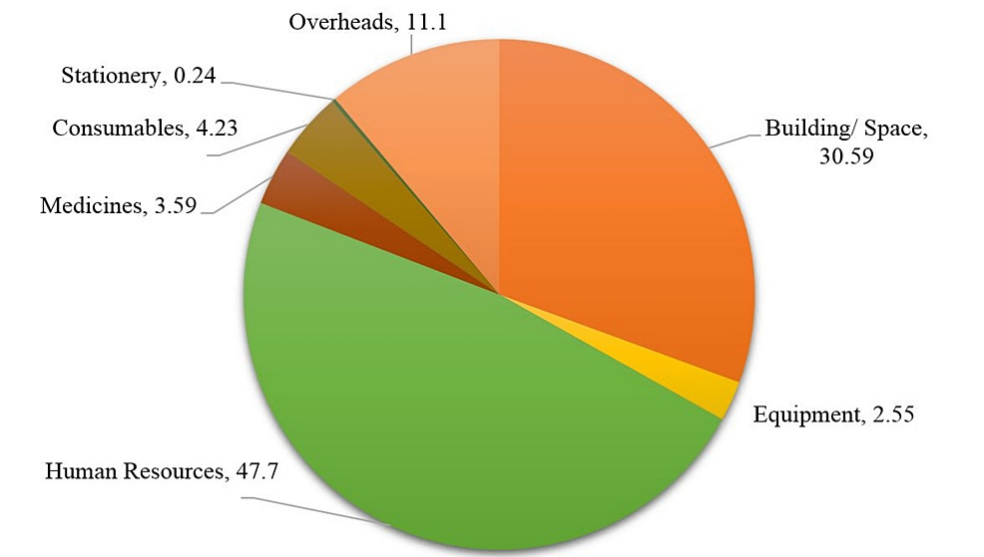
Proportion of various cost heads in the total cost of intranatal care service (in %)

The annual cost of intranatal services for FY 2020-21 was INR 149.01 million (USD 1.98 million) spent in various heads (Table 1). The capital and recurrent costs were 33.14% and 66.86% of the total costs, respectively. Human resources were the predominant cost, accounting for 47.7% of the total, followed by building/space and overhead costs, accounting for 30.59% and 11.1%, respectively (Table 1 and Figure 4).

**Table 1:** Cost of intranatal care services per type of resources and cost centers (FY 2020-2021) FY: Financial year

Cost Head	Pre-Delivery Room: Cost in INR (USD)	Clean Labor Room: Cost in INR (USD)	Septic Labor Room: Cost in INR (USD)	Intensive Care Unit: Cost in INR (USD)	Operation Theatre: Cost in INR (USD)	Laboratory: Cost in INR (USD)	Radiology: Cost in INR (USD)	Total Cost in INR (USD)
Capital Items								
Building/Space	9,617,962 (128,325)	11,669,186 (155,693)	11,350,107 (151,436)	1,230,734 (16,421)	10,666,365 (142,313)	729,324 (9,731)	319,079 (4,257)	45,582,758 (608,176)
Equipment	102,595 (1,369)	315,384 (4,208)	433,178 (5,780)	353,382 (4,715)	1,246,336 (16,629)	577,570 (7,706)	771,360 (10,292)	3,799,805 (50,698)
Total Capital	9,720,557 (129,694)	11,984,570 (159,901)	11,783,284 (157,215)	1,584,116 (21,136)	11,912,701 (158,942)	1,306,894 (17,437)	1,090,440 (14,549)	49,382,562 (658,873)
Recurrent Items								
Human Resources	16,134,866 (215,275)	8,245,130 (110,008)	11,159,356 (148,891)	8,316,208 (110,957)	20,399,588 (272,176)	5,899,532 (78,713)	924,023 (12,329)	71,078,703 (948,348)
Medicines	786,381 (10,492)	1,160,848 (15,488)	1,401,577 (18,700)	705,068 (9,407)	1,294,586 (17,273)	1,070 (14)	-	5,349,529 (71,375)
Consumables	556,573 (7,426)	1,048,223 (13,986)	1,166,912 (15,569)	705,959 (9,419)	2,127,773 (28,389)	584,307 (7,796)	113,458 (1,514)	6,303,206 (84,099)
Stationery	68,236 (910)	80,824 (1,078)	90,838 (1,212)	19,312 (258)	72,956 (973)	20,742 (277)	4,721 (63)	357,629 (4,772)
Overheads	1,885,597 (25,158)	2,684,495 (35,817)	2,537,286 (33,853)	793,936 (10,593)	7,393,526 (98,646)	980,841 (13,087)	264,645 (3,531)	16,540,327 (220,685)
Total Recurrent	19,431,652 (259,262)	13,219,520 (176,378)	16,355,969 (218,225)	10,540,483 (140,634)	31,288,430 (417,457)	7,486,493 (99,887)	1,306,847 (17,436)	99,629,394 (1,329,278)
Total (overall)	29,152,209 (388,955)	25,204,089 (336,279)	28,139,254 (375,440)	12,124,599 (161,769)	43,201,131 (576,399)	8,793,388 (117,323)	2,397,286 (31,985)	149,011,957 (1,988,152)

**Figure 5:**
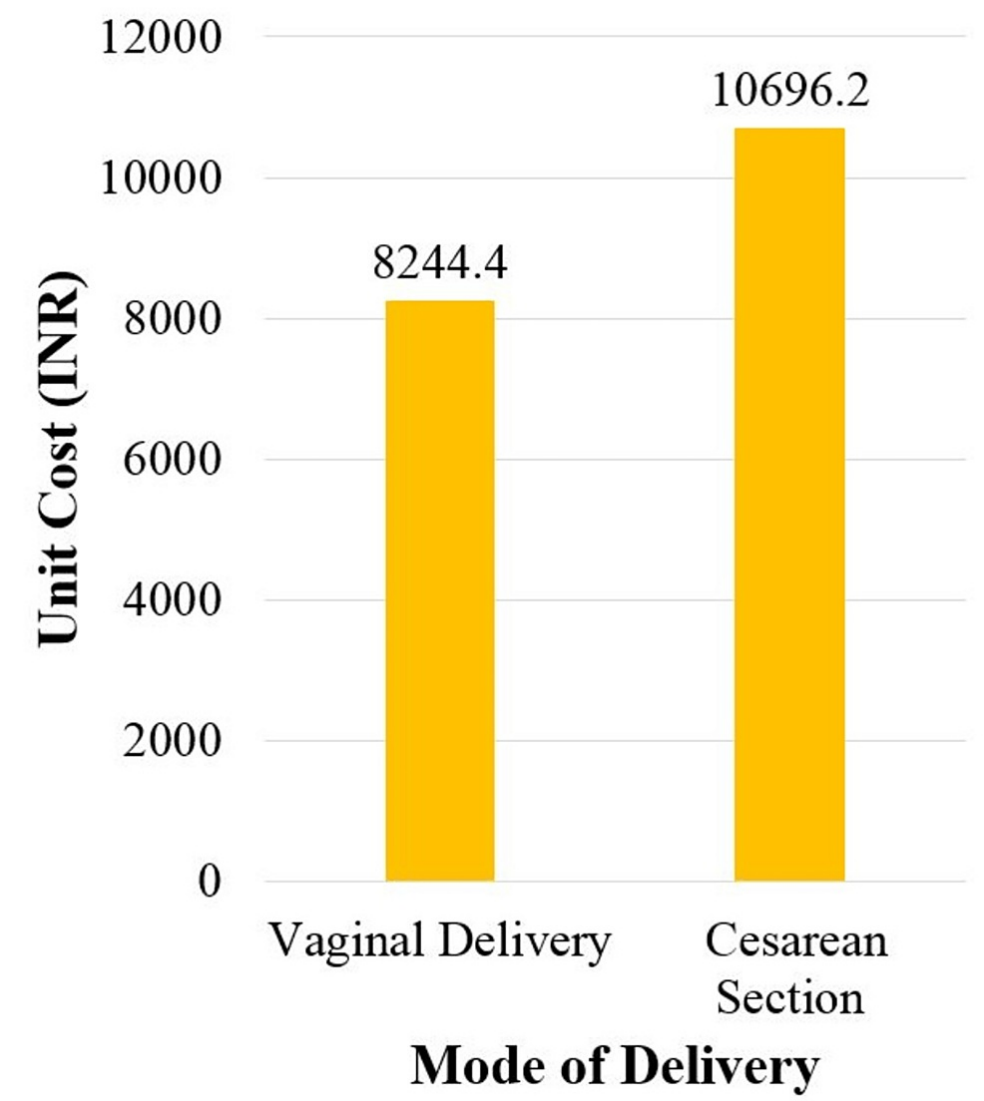
Comparison of unit cost for vaginal delivery and caesarean section (FY 2020-2021) FY: Financial year

The unit costs for providing intranatal care at a tertiary-level hospital were INR 12,717 (USD 169.7) per institutional delivery, INR 8,244.4 (USD 109.9) per vaginal delivery, and INR 10,696.2 (USD 142.7) per caesarean section (Table 2 and Figure 5).

**Table 2:** Unit cost of intranatal care at various cost centers (FY 2020-2021) # Inclusive of women in labor who absconded * Inclusive of cost for women who did not deliver but took the services and then absconded **Unit cost per constituent of various diagnostics FY: Financial year

Cost Center	Total Cost INR	Output	Unit	Unit cost INR (USD)
Pre-Delivery Room	29,152,209	11,961^#^	Beneficiary	2,437.3 (31.42)
Clean Labour Room (CLR)	25,204,089	4,398	Beneficiary	5,730.8 (76.5)
Septic Labour Room (SLR)	28,139,254	2,649	Beneficiary	10,622.6 (141.7)
ICU	12,124,599	653	Beneficiary	9,283.8 (123.9)
Operation Theatre	43,201,131	5,630	Cases/Surgeries	7,673.4 (102.38)
Total Laboratory**	8,793,388	115,255**	Number of investigations	76.3 (1.02)
Radiology	2,397,286	4,707	Beneficiary	509.6 (6.8)
Total	149,011,957	11,718	Beneficiary	12,717* (169.7)*

**Figure 6:**
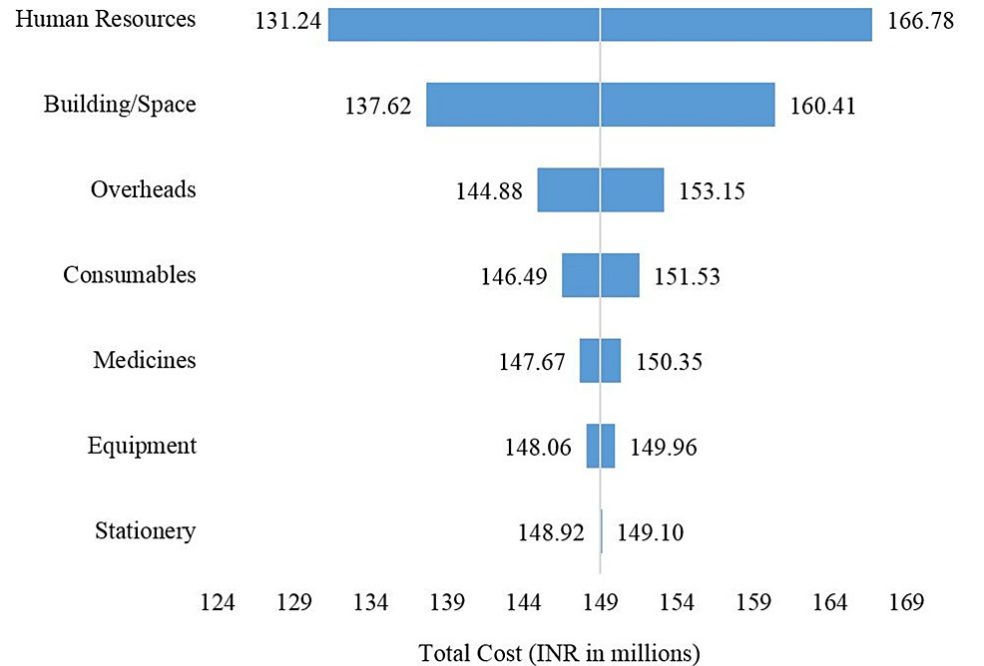
Tornado diagram showing sensitivity analysis of the total cost to ± 25% variation in various cost heads of intranatal care

At 25% variation of various types of costs of intranatal care, the sensitivity analysis showed that the total cost was most sensitive to the cost of human resources, followed by building/space, overhead, consumables, medicine, equipment, and stationery (Figure 6).

## Discussion

Comparisons of various studies across the globe have been made after currency changes to the USD and adjustments for inflation to the base year 2020 [21]. The present study reported the unit cost for vaginal delivery and caesarean section as USD 109.9 (INR 8244.4) and USD 142.7 (INR 10696.2), respectively. In the Indian context, a few multi-centric studies have reported the cost of institutional delivery at the secondary level of healthcare. Prinja et al. reported the cost per institutional delivery as USD 62.9 (95% CI 43.8-95.8) which was lower than that of the present study, maybe because they calculated it for the secondary level of healthcare [17]. Singh et al. reported the mean cost for vaginal delivery and caesarean delivery as USD 149 (min-max: 53 to753) and USD 226 (min-max: 79-529), respectively, which were higher than that observed in the present study, maybe because they included the cost of postnatal stays in their calculations, whereas, in the present study, postnatal costs were not included and the focus remained exclusively on intranatal services [18]. Under health insurance schemes such as the *Chiranjeevi Yojana* [10] and the *Ayushman Bharat Pradhan Mantri-Jan Aarogya Yojana* (AB PM-JAY) [8,9], the Rajasthan government pays INR 11,500 (USD 153.4) and INR 14,100 (USD 188.1) per caesarean delivery, respectively. These reimbursement rates are slightly higher than those in the present study, as they are also inclusive of the average postnatal stay of the beneficiaries.

In the present study, salaries for human resources were the predominant cost (47.7%), which is well in resonance with the findings of other costing studies conducted in India [17,18]. The 12th five-year plan report by the working group on tertiary care institutions also reported that salaries accounted for as much as 70% of the total health budget [22]. Internationally, human resources were also reported as the major cost driver [23,24].

Internationally, studies have been discussed per the income levels of the respective countries. In other lower-middle-income countries (LMIC), a study at a tertiary-level public hospital in Pakistan by Khan et al. reported the average cost for vaginal delivery and caesarean section as USD 42.9 and USD 173.4, respectively [23]. The former cost was lower than that of the present study, while the latter was higher, which may be due to intercountry hospital variations and variations in operation theater charges. Another study in Bangladesh (LMIC) by Zeng et al. utilized both top-down and bottom-up methodology and reported substantially lower costs than the present study [25]. Apart from intercountry variations, this may be due to differences in the level of the health facility as well as the type of administrative control, which was under a non-government organization (NGO). Löfgren et al. reported the cost of a caesarean section in a public and private hospital as USD 67.5 and USD 73.4, respectively, in Uganda, a low-income country (LIC) [26]. A study in Brazil (upper-middle-income countries (UMIC)) by Entringer et al. reported the mean cost of vaginal delivery and elective caesarean delivery as USD 190.39 (USD 137.99 to USD 215.83) and USD 262.37 (USD 153.76 to USD 357.15) respectively, utilizing micro-costing and top-down techniques [24]. Duro-Gómez et al. of Spain [27] and Fawsitt et al. of Ireland [28] also reported higher costs, which may be because these studies were conducted in high-income countries.

The present study's caesarean section rate was more than 50%, which is much higher than the average ideal caesarean section rate of 10% to 15% recommended by WHO [29] and also higher than the 15.3% reported by the National Family Health Survey-5 (NFHS-5) for Rajasthan [13]. These higher rates are probably because the hospital is an apex hospital where the maximum cases are referred; nevertheless, the indication of caesarean section should be monitored. 

Recommendations

Recommendations include monitoring the indication of a caesarean section, whether elective or emergency, which may alter the caesarean section rate and cost. Also, the costs and effects should be viewed side by side to inform better financial and policy decisions at the local administrative level. It is recommended to conduct such studies in the future to generate more evidence regarding various types of costs related to intranatal care services, along with cost-effectiveness and cost-benefit analyses of intranatal care services, for better evidence-based decision-making. 

Limitations

Although the present study was conducted with a well-designed schedule, a few limitations at the micro-level of cost analysis were inevitable. The effects of COVID-19 were at its peak during the financial year 2020-2021, which may affect the various types of related costs. Limitations arising from missing real cost data for any equipment or consumables due to poor record maintenance were overcome by taking proxy data measures or market prices of FY 2020-2021. 

## Conclusions

The present study concludes that the unit cost of a caesarean section was 1.3 times higher than the unit cost of vaginal delivery at a tertiary-care public hospital in Rajasthan, India. The maximum proportion of the cost of intranatal care at the tertiary-care public hospital was for 'human resources, followed by 'building/space'. The total cost of intranatal care was most sensitive to 'human resources, followed by 'building/space' among the various cost heads. 

These findings may be helpful to planners at the Health Department of Rajasthan and also for the revision of reimbursement rates for intranatal care services under various public sector insurance schemes. Also, this study will help in the judicious utilization of limited resources and may serve as a basis for future health economic studies. 
